# Eating disorders in biological males: clinical presentation and consideration of sex differences in a pediatric sample

**DOI:** 10.1186/s40337-018-0226-y

**Published:** 2018-11-26

**Authors:** Jennifer S. Coelho, Tiffany Lee, Priscilla Karnabi, Alex Burns, Sheila Marshall, Josie Geller, Pei-Yoong Lam

**Affiliations:** 1 Provincial Specialized Eating Disorders Program for Children & Adolescents, British Columbia Children’s Hospital, Box 178, 4500 Oak St., Vancouver, BC V6H 3N1 Canada; 2 Department of Psychiatry, University of British Columbia, Vancouver, BC Canada; 3 School of Social Work, University of British Columbia, Vancouver, BC Canada; 4 Division of Adolescent Health & Medicine, Department of Pediatrics, University of British Columbia, Vancouver, BC Canada; 5 Eating Disorders Program, St. Paul’s Hospital, Vancouver, BC Canada

**Keywords:** Males, Pediatric, Eating disorders, Anorexia nervosa, Sex differences

## Abstract

**Background:**

The growing body of research on eating disorders among male adolescents reveals some sex differences in clinical presentation. The current study set out to replicate and extend recent research on the clinical and medical characteristics of male youth with eating disorders, and examine sex differences between biological males and females in a tertiary pediatric eating disorder treatment setting.

**Methods:**

A retrospective chart review was conducted with all biological males who were admitted to the Eating Disorders Programs at British Columbia Children’s Hospital (2003–2015) or the Looking Glass Residence (2011–2015). Clinical data, including demographics, percentage of median body mass index (% mBMI), and psychiatric diagnoses, were recorded along with medical data (i.e., vital signs, basic biochemistry investigations, and bone mineral density). A comparison group of females with eating disorders who received treatment at British Columbia Children’s Hospital in the inpatient or outpatient streams (2010–2015) were included, to examine sex differences with males who were admitted during the same period.

**Results:**

A total of 71 male youth were included in the chart review. Males had significant medical complications, with 26.5% of the sample presenting with a heart rate of less than 50 beats per minute and 31.4% presenting with a bone mineral density z-score for the lumbar spine ≤ − 1. Sex differences between the subset of males who were treated between 2010 and 2015 (*n* = 41) and the females (*n* = 251) were examined. Females were more likely than were males to have a diagnosis of anorexia nervosa or bulimia nervosa, and to be underweight (< 95% mBMI) at admission. Males were younger than females, but no differences emerged in the duration of the eating disorder symptoms. No sex differences emerged relating to medical instability (e.g., bradycardia).

**Conclusions:**

A large proportion of male children and youth with eating disorders are medically compromised at admission. Males were younger than females, and were less likely than females to have a diagnosis of anorexia nervosa or bulimia nervosa. Males who were underweight at admission had also lost a lower percentage of body weight in comparison to females. The current study replicates previous sex differences reported in pediatric samples.

## Plain English Summary

There is limited research on the presentation of males with eating disorders. The overwhelming majority of individuals presenting to specialized eating disorder treatment centers are female; however, there are concerns that eating disorders in males are underdiagnosed and misunderstood. The current study set out to examine the clinical characteristics of a large sample of males who presented for treatment between 2003-2015 at a specialized treatment program for eating disorders. We compared a group of females who had received treatment at this program between 2010-2105 with males who were admitted during the same period. The males who were treated during this period had significant medical complications, with approximately one quarter of the sample presenting with very low heart rate and one third presenting with a low bone mineral density. Sex differences among adolescents with eating disorders also emerged: males were younger than females, and had a younger age of onset than did females. Males were also less likely than females to have a diagnosis of anorexia nervosa or bulimia nervosa. The current diagnostic classification system for eating disorders may not capture some of the eating- and weight-related concerns experienced by males, given the larger proportion of atypical and other eating disorder diagnoses in males.

## Background

The published literature on eating disorder presentation in adolescent males has been growing over the past several years. In 2012, a special issue on males and eating disorders was published in *Eating Disorders: The Journal of Treatment and Prevention.* Male eating disorders were highlighted to be “under-diagnosed, undertreated, and misunderstood” by many clinicians (p. 346), [1]. A small body of literature on the clinical characteristics of males with eating disorders had been established over the past several decades, prior to the publication of this special issue. In one of the largest samples of males with eating disorders (135 adult males), bulimia nervosa was the most common diagnosis, with reports of long delays in seeking treatment [2]. Delays in referrals of males with eating disorder symptoms to specialist services were also highlighted in a sample of adolescents [3].

In contrast to the prevalence of bulimia nervosa in the large adult male sample reported by Carlat and colleagues [2], anorexia nervosa (AN) was the most common diagnosis in a sample of adolescent males, followed by atypical eating disorder diagnoses [4]. In line with this presentation, a sample of 10 males with AN between the ages of 9–22 who were treated in a tertiary hospital setting all presented with very low weight (less than 80% of suggested body weight) [5]. In contrast, in a small sample of males with an early onset eating disorder (i.e., onset at or before age 13), the majority of patients did not meet criteria for AN despite significant weight loss and medical instability [6]. A recent review suggests that the current diagnostic classification scheme for full threshold eating disorders, including AN and bulimia nervosa (BN), may have less applicability for males, given the inclusion of the pursuit of thinness as a prominent feature of the diagnostic criteria [7]. Muscularity-related concerns are one of the purported features of eating disorder concerns in males, which are not currently accounted for in the diagnostic criteria for eating disorders [7]. Muscularity concerns emerged as one of four symptom classes of eating disorder symptom patterns (along with binge-eating/purging, body image, and a mostly asymptomatic group with some muscularity concerns) in a large sample of male youth [8]. The high prevalence of excessive exercise that has been reported in males with eating disorders (e.g., [9]) may reflect the high level of muscularity concerns in some males.

Younger, pre-adolescent patients who present to eating disorder treatment services are more likely to be male than are older adolescents [10, 11]. One of the challenges of interpreting these results are that males appear to be more reluctant to seek treatment than females, due to perceived stigma about seeking help [12]. The higher rates of younger males in treatment that have been reported in the literature may reflect a decrease in help-seeking behaviour for older males with eating disorders (rather than a difference in prevalence of eating disorders across children and adolescents). The higher rate of younger male patients, however, suggests a particular relevance of studying male eating disorders in pediatric settings. One of the largest pediatric samples of males studied to date examined the medical and psychological characteristics of male adolescents with eating disorders [13]. Norris and colleagues [13] described a total of 52 male adolescents with eating disorders between the ages of 10 to 17, who had received treatment at a tertiary eating disorders program in Canada. The most common diagnosis in this group was Eating Disorder Not Otherwise Specified (EDNOS; 52% of the sample), followed by AN and BN. Food restriction was reported by the overwhelming majority of the sample, with over-exercise, binge episodes, vomiting and laxative use reported by a subset of the males. A notable proportion of males for whom bone mineral density z-scores were available were found to have osteopenia (31%) or osteoporosis (8%).

Several male-specific studies have recently emerged, which further examined the medical and clinical characteristics of males with eating disorders. Vo and colleagues [14] reported on a sample of 33 male outpatients (ages 11–25), with a focus on medical characteristics. Of note, a large proportion (51.5%) of the sample met criteria for admission for medical stabilization due to low heart rate and large orthostatic shifts in heart rate (more than 20 beats per minute) at presentation. Although males presented with a mean of 88% median body mass index (mBMI), there was an average weight loss of 20% of initial body weight. Male adolescents with eating disorders present at a higher percentage of their suggested body weight than females [15]. Similarly, adult males with AN have a higher body mass index (BMI) at admission than do females [16]. There have been criticisms of the use of BMI in examining sex differences in anthropometric measures, due to sex- and age-related differences in body composition (i.e., body fat versus muscle mass) [17]. Nagata and colleagues have employed dual-energy x-ray absorptiometry (DXA) scans to examine sex differences in body composition, and report that there are deficits in fat mass and lean body mass in both adolescent males and females [18]. However, there are not currently guidelines recommending the use of DXA scans to evaluate body composition in males [18].

Woodside and Kaplan [19] report that the clinical presentation of males and females who were admitted to a day treatment program was very similar, and that males could be effectively treated in a group setting comprised predominantly of females. However, some mixed findings are emerging with respect to sex differences in the clinical presentation of males and females. Several studies suggest no sex differences in admission age (e.g., [15, 20–25]), while one study with an adolescent sample reported females were older than males [26]. Adult males have been reported to have a later age of onset [16, 27], whereas studies with adolescent samples report a younger age of onset in males [26, 28]. Across studies, some conflicting findings between the pediatric and adult literature have therefore emerged with respect to age at admission and age of onset. With the exception of the two studies on sex differences in adolescents [26, 28] all studies were conducted prior to the publication of the 5th edition of the Diagnostic and Statistical Manual (DSM-5) [29] and therefore do not consider sex or age-related differences in the prevalence of avoidant/restrictive food intake disorder (ARFID). Some of the inconsistencies across the literature can potentially be attributed to low power due to small sample sizes; however, there are a growing number of large studies (sample sizes greater than 50 males; (e.g., [20–23, 26, 28]).

Further discrepancies in the literature arise when considering eating disorder diagnostic presentation. Some researchers have reported no sex differences in eating disorder diagnostic categories [22, 24], while others have reported that males are less likely than are females to present with a diagnosis of AN or BN or more likely to present with an atypical or other eating disorder (e.g., [26, 28]). Children and adolescents who were diagnosed with ARFID were also more likely to be male in comparison to those diagnosed with AN or BN [30]. Inconsistencies also arise in examination of sex differences in symptom presentation. While some studies report no differences in eating disorder symptom presentation [26, 27], others report that males have lower levels of laxative abuse [20, 31] and vomiting [28] than do females with eating disorders. Males have been reported to be more likely to have over-exercise as an eating disorder symptom than are females (e.g., [3]), while a more recent study with a larger sample size reported no sex differences in the prevalence of excessive exercise [28]. Researchers have reported the absence of sex differences in medical instability (e.g., bradycardia and hypotension) (e.g., [28]).

In addition to developmental considerations of patient samples, another possible factor that could account for differences in clinical presentation across studies is the intensity of the treatment setting. Whereas some studies investigating presentation of males have focused on individuals presenting to an outpatient clinic (e.g., [14, 26]), other studies have been conducted at centers that offer a continuum of care (including outpatient, day treatment, and inpatient services) (e.g., [28]). A report from our group revealed that among a sample of male inpatients, the predominant diagnosis was AN (restrictive or binge-eating/purging subtype), with 82.6% of the sample receiving this diagnosis [32]. The high prevalence of AN reported by Coelho and colleagues [32] exceeds that reported by Norris and colleagues [13], in which 42% of males were diagnosed with AN. Similarly, Shu and colleagues [28] reported lower levels of AN diagnoses in their sample, at 34% of males (and 38.1% of females). The fact that Coelho and colleagues [32] focused exclusively on an inpatient sample may account for some of the differences in the prevalence of AN across studies. Over the past decade there has been an increasing focus of outpatient services as the first-line intervention for eating disorders, including family-based treatment [33]. Furthermore, some of the youth who have been referred to treatment in a pediatric eating disorders setting may have lost weight and be experiencing significant medical complications associated with eating disorders, but may not yet meet criteria for AN. Atypical eating disorders may be more common in those who are admitted to lower intensity treatment for eating disorders (i.e., outpatient services), as these individuals may be more medically stable or have lost less weight than those admitted to a higher level of care. Individuals with other eating disorders may also be more likely to be admitted to outpatient services. For example, those with a diagnosis of ARFID are less likely than those with AN to present with low heart rate and hypotension [34]. The current study therefore set out to examine diagnostic differences across males who were treated in outpatient versus intensive (day treatment/inpatient) services.

### Aims and objectives

The goal of the current research is to replicate and extend research on the clinical features of male youth with eating disorders, and examine sex differences in pediatric eating disorders. A retrospective chart review was conducted to achieve this goal. We endeavored to extend the report by Vo and colleagues [14] on clinical and medical characteristics of male youth. We hypothesized that males admitted to outpatient would be more likely than those admitted to a more intensive treatment (i.e., day treatment and/or inpatient) to have an eating disorder diagnosis other than AN or BN. We further set out to replicate previous reports of sex differences in male and female adolescents [26, 28]. We therefore expected a higher prevalence of diagnoses other than AN or BN in males than in females, and a younger age of onset than females. Given the diversity of youth presenting to our service, we also set out to examine whether males would be more likely to come from an ethnic minority group than would females, as reported by Kinasz and colleagues [26]. We also conducted exploratory analyses to assess sex differences on key medical and clinical characteristics, including presence of eating disorder symptoms, medical stability (bradycardia, orthostatic shift, and bone mineral density z-score), and weight-related presentation at admission (i.e., % mBMI at admission, and percentage of weight loss at presentation). Bradycardia and orthostatic shift were classified based on admission criteria published by the Society for Adolescent Health and Medicine [35].

## Methods

### Participants

Inclusion criteria for the study were any biological males (based on assigned sex at birth) who were assessed and admitted to treatment at either British Columbia (BC) Children’s Hospital (Provincial Specialized Eating Disorders Program for Children and Adolescents) between January 2003 and July 2015^1^ or the Looking Glass Residence (formerly Woodstone Residence) between April 2011 (when the residence opened) and July 2015. Data from the first admission during the study period were recorded. Admission to the program was defined as attending at least one medical or therapy appointment (for outpatients), or admission to one of the more intensive treatment programs (i.e., having at least 1 day of admission to day program, inpatient, or residential treatment).

BC Children’s Hospital offers outpatient treatment, a day treatment program, and inpatient treatment. Youth (and their families) attend an initial assessment in the program, and the level of care (outpatient, day treatment or inpatient) is recommended by the multi-disciplinary assessment team, in collaboration with youth and their family, after evaluation of medical stability, symptom severity, and other characteristics of the patient and family (including past treatment history and availability of parents/caregivers to provide meal support). The assessment team includes a pediatrician, nurse, and either a psychiatrist or psychologist. BC Children’s Hospital is the only tertiary hospital-based program in the province. Patient and family readiness for treatment and recovery are considered when evaluating an appropriate level of care at assessment; however, some youth may be certified for involuntary treatment given the potential for permanent medical consequences of eating disorders.

Admission criteria for outpatient treatment (e.g., family-based treatment) include medical and psychiatric stability, willingness to engage in treatment, and availability of parent(s) or caregiver(s) to provide meal support. Admission criteria for day treatment includes medical stability (i.e., not meeting hospital admission criteria [35]), no acute safety-related concerns, ability and willingness of youth and family to engage in treatment, significant life interference, and need for tertiary level services. Inpatient treatment would be recommended for individuals who have failed to make sufficient progress in a lower level of care, and/or whose symptoms require intensive treatment. Youth who are not medically or psychiatrically stable may be admitted to a medical or psychiatric bed for stabilization prior to transfer to the inpatient Eating Disorders Program. Given the wide geographical area served by the hospital setting, inpatient treatment may be recommended for children and adolescents who do not have eating disorders services in their local community, and whose family are not able to support their child to attend a lower level of care.

The Looking Glass Residence offers treatment for individuals with eating disorders between ages 16–24 in a residential setting, with 24-h support provided by a multi-disciplinary team of health professionals (including psychiatrists, nursing, allied health professionals, and a nurse practitioner). Admission criteria include medical and psychiatric stability. Looking Glass Residence is a voluntary recovery-focused treatment program, which offers an intensive 12-week program of structured therapy in a home-like setting. Details of this program are available in from the authors, and are detailed in: Williams KD, O’Reilly C, Coelho JS: Residential treatment for eating disorders in a Canadian treatment Centre: clinical characteristics and treatment experiences of residents, submitted.

Information regarding participants’ eating disorder and other psychiatric diagnoses was recorded from documentation by staff psychiatrists or psychologists, in accordance with the 4th edition of the Diagnostic and Statistical Manual of Mental Disorders (DSM-IV-TR) [36] criteria, or DSM-5 [29] criteria for those admitted after May 2013. A retrospective review of the charts of biological males who met inclusion criteria was undertaken. A standardized data extraction manual was used to ensure consistency among members of the research team in data entry and checking. Study data were collected and managed using REDCap (Research Electronic Data Capture) tools hosted at BC Children’s Hospital Research Institute. REDCap is a secure, web-based application designed to support data capture for research studies [37].

To facilitate an analysis of sex differences, a comparison group of biological females (based on assigned sex at birth) who were admitted to treatment was analyzed. Given the small number of males admitted to the Looking Glass Residence during the study period, and the differences in admission criteria between hospital-based and residential treatment, the comparison group was limited to those who were admitted to the Eating Disorders Program at BC Children’s Hospital. Data from the first admission of biological females with eating disorders who were admitted to the inpatient unit (March 2010–June 2015^2^) or outpatient family-based therapy (January 2010 – July 2015^3^) was available as part of other on-going research in the Eating Disorders Program, and was included as a convenience sample for the female comparison group.

Procedures in this study were reviewed and approved by the Research Ethics Board at the Children’s and Women’s Health Center of British Columbia (see Declarations section for details).

## Results

### Clinical and demographic characteristics of male youth

Charts were available for 71 male youth, 68 of whom had their first admission at BC Children’s Hospital during this period, and 3 who were admitted to the Looking Glass Residence. One male who was first admitted to BC Children’s Hospital had a subsequent admission to the Looking Glass Residence during the study period; however, only data from the first admission were analyzed. Pairwise deletion of data was performed, in which cases that did not have data for a particular variable were excluded from that analysis. The sample size for each analysis is indicated for variables for which there was missing data.

Male youth had an average age of 14.8 years (SD = 2.8, range = 9–24 years). Due to a non-normal distribution for the duration of eating disorder symptoms, median and interquartile range (IQR) were evaluated. Duration of eating disorder symptoms was calculated based on the number of months that had elapsed between the first reported onset of eating disorder symptoms and the date of assessment. For cases in which a range in the time frame was given for the onset of symptoms (e.g., a season, or school grade), the midpoint of the time period was recorded. The median duration of symptoms prior to admission was 11 months (IQR = 18.0, range = 2–148; *N* = 69). Participants were from a diverse ethnic background, including: Caucasian (*n* = 27, 38.0%), Asian (including South Asian, West Asian, and Chinese backgrounds; *n* = 15, 21.1%), mixed background (*n* = 5, 7.0%), Latin American (*n* = 2, 2.9%), Aboriginal (*n* = 1, 1.4%), and Pacific Islander (*n* = 1, 1.4%). Information on ethnic background was not available for 20 participants (28.2%). One of the males reported questioning his gender identity at assessment, but did not have a formal diagnosis of gender dysphoria at admission. Demographic information from the sample is presented in Table 1.

**Table 1:** Demographic and presenting characteristics of male youth

Living Arrangements (*n* = 71)	
Living with both parents (biological or adoptive/foster parents)	45 (63.4%)
Living with mother (biological or adoptive/foster parent)	16 (22.5%)
Living equally in two homes (parents or other caregivers)	5 (7.0%)
Living with mother and a step-parent	3 (4.2%)
Living with other caregivers (e.g., grandparents)	2 (2.8%)
Prior Eating Disorder Treatment (*n* = 71)	
Community hospital admission (medical stabilization)	27 (38.0%)
Outpatient eating disorder treatment	29 (40.8%)
Inpatient admission to a specialized eating disorders program	1 (1.4%)
Day treatment/residential treatment admission	0 (0%)
Certification for Involuntary Treatment (*n* = 66)	
Voluntary admission	54 (81.8%)
Certification for part or all of admission	12 (18.2%)
Vegetarianism (*n* = 66)	
Partial Vegetarian (most meals are vegetarian)	3 (4.5%)
Full vegetarian	5 (7.6%)
Vegan	1 (1.5%)
Not Vegetarian	57 (86.4%)
Self-harm and Suicidality (*n* = 71)	
Current self-harm behaviours	5 (7.0%)
Past history of self-harm behaviours	5 (7.0%)
Previous suicide attempt	5 (7.0%)
History of abuse	
Physical abuse reported at assessment (*n* = 69)	4 (5.8%)
Sexual abuse reported at assessment (*n* = 68)	1 (1.5%)
Co-occurring psychiatric diagnoses at assessment (*n* = 71)	
Major depressive disorder	10 (14.1%)
Other mood disorder (including not otherwise specified/unspecified, and persistent depressive disorder/dysthymia)	2 (2.8%)
Generalized anxiety disorder	3 (4.2%)
Social anxiety disorder	3 (4.2%)
Panic disorder	2 (2.8%)
Other anxiety disorder (including not otherwise specified/unspecified anxiety disorder, selective mutism, and specific phobia)	11 (15.5%)
Obsessive-compulsive disorder	3 (4.2%)
Attention-deficit/hyperactivity disorder	3 (4.2%)
Autism spectrum disorder	2 (2.8%)
Substance-related disorder	1 (1.4%)

For the youth who received treatment at BC Children’s Hospital, the majority were first admitted to outpatient treatment (*n* = 35, 51.5%), 28 (41.2%) were admitted to inpatient treatment, and 5 (7.4%) were admitted to day treatment. Several youth who were admitted to the outpatient stream had shared care with other treatment programs at BC Children’s Hospital (i.e., an inpatient mental health admission for child or adolescent psychiatry, *n* = 5, 7.3%), or with other community-based mental health teams (*n* = 2, 2.9%) for support with co-occurring mental health concerns. A majority of the males (*n* = 44, 62.0%) had received previous treatment for an eating disorder prior to admission to the Eating Disorders Program (see Table 1 for details). Previous treatment was reported at assessment based on parent or youth report and/or information included in the referral, and was defined as admission for treatment of an eating disorder to: a community hospital, specialized eating disorder inpatient or day treatment program, or outpatient services for an eating disorder (e.g., secondary service programs or private therapist providing eating disorder treatment). A subgroup of the 68 youth who were admitted to BC Children’s Hospital had been treated in another department immediately prior to their transfer to the eating disorders program, including a medical stabilization admission (*n* = 12, 17.6%), admission to inpatient psychiatry or psychiatric emergency (e.g., for concerns about psychiatric safety or suicidality *n* = 5, 7.3%), or a visit to emergency without subsequent admission (*n* = 1, 1.1%).

The most common eating disorder diagnosis was AN (restrictive subtype, *n* = 27, 38%; binge-eating/purging subtype, *n* = 9, 12.7%), followed by EDNOS/other specified feeding or eating disorder (OSFED) with primary symptoms of restriction (*n* = 20, 28.2%), EDNOS/OSFED with symptoms of binge-eating and/or purging (*n* = 3, 4.2%), and BN (*n* = 2, 2.8%). Three males (4.2%) met the criteria for ARFID. Additional diagnoses included failure to thrive (*n* = 1, 1.4%) and food avoidance emotional disorder (*n* = 3, 4.2%) under DSM-IV-TR [36], with symptoms that would likely meet DSM-5 [29] criteria for ARFID. Finally, three males (4.2%) reported symptoms of disordered eating that did not meet full criteria for an eating disorder diagnosis (or were on a differential diagnosis with EDNOS/OSFED) but were causing significant impairment in well-being or functioning, and therefore required specialized treatment.

To examine the hypothesis that males who received outpatient treatment would be more likely than those in an intensive treatment to present with a diagnosis other than AN or BN, eating disorder diagnoses were categorized into one of three groups: (1) diagnosis of AN or BN; (2) atypical AN or BN (including EDNOS with restrictive or binge-eating/purging symptoms); and (3) other eating disorder diagnoses (e.g., unspecified eating disorder, purging disorder, ARFID). We selected only those who were admitted to BC Children’s Hospital (given the limited sample size of those who received residential treatment, and the differences with residential treatment admission criteria, participants from the Looking Glass Residence were excluded). There was a significant difference in diagnostic classification across treatment intensity (outpatient versus day treatment/inpatient), *χ*2 (2, *N* = 68) = 17.05, *p* ≤ .001. Z-tests (with Bonferroni correction applied) to assess differences in column proportion suggested that there were fewer males in outpatient with a diagnosis of AN or BN (*n* = 10, 28.6%) than in the inpatient or day treatment programs (*n* = 25, 75.8%). There were more individuals with an “other” eating disorder diagnosis in outpatient (*n* = 11, 31.4%) than in inpatient/day treatment (*n* = 1, 3.0%). No differences in treatment intensity emerged for those with atypical eating disorders who were treated in outpatient (*n* = 14, 40.0%) versus inpatient/day treatment (*n* = 7, 21.2%).

Eating disorder symptoms reported at assessment (by either the youth or the parent) were recorded. Eating disorder symptoms were marked as present if there was a lifetime history of the symptom. Restriction was the most prevalent eating disorder symptom reported at assessment, with nearly all of the males (*n* = 69, 97.2%) restricting their intake. Excessive exercise was also a common symptom, with 70.4% (*n* = 50) of the youth presenting with symptoms of excessive exercise. Excessive exercise was defined as activity that was aimed at controlling weight or shape, and that was significant in its duration, frequency and/or intensity (including exercising despite injury), and/or interferes with activities, as in the DSM-5 ([29], p., 346). A subgroup of males reported regular episodes of binge-eating (*n* = 20, 28.2%) and purging through vomiting (*n* = 14, 19.7%) and/or laxative use (*n* = 2, 2.8%).

The percentage of median BMI (% mBMI) was calculated for youth ages 19 and under (*n* = 68), according to reference values from the World Health Organization [38]. At admission, the mean percentage mBMI of youth was 87.5% (*SD* = 12.2). The majority of males were underweight (< 95% mBMI) at admission (*n* = 51, 75% of sample). There was a significant increase in weight over the course of admission, *t*(45) = 9.6, *p* ≤ .001, *d* = 1.3; *M*_admission_ = 82.2, *SD* = 7.4; *M*_discharge_ = 94.3, *SD* = 10.3). Length of treatment was also investigated. Due to a non-normal distribution of length of treatment, median and IQR were investigated. The median length of treatment for the 61 males who completed treatment during the study period and for whom discharge data was available, was 90 days (IQR = 124, range = 1–661 days).

A large proportion of the sample (*n* = 47, 66.2%) had symptoms of a co-occurring psychiatric disorder at assessment, and received either a full diagnosis of a co-occurring psychiatric disorder, or symptoms of a co-occurring psychiatric disorder were present but were on a differential diagnosis and therefore were not assigned (for example, due to malnutrition at the time of assessment). For details of the diagnoses for which participants met full criteria, see Table 1.

Biochemistry results of the male sample are presented in Table 2. Approximately one third of the sample for whom transaminases were available (alanine aminotransferase; *n* = 46) presented with levels that were out of range. A portion of the sample (13.3%) for whom testosterone levels were available (*n* = 30) had levels that were below the expected range. Medical characteristics of the male youth are presented in Table 3. Indices of medical compromise included heart rate, with 26.5% of the sample (*n* = 68) presenting with bradycardia (i.e., a heart rate of below 50 beats per minute), and 33.3% (20 of sample size of 60) presenting with significant orthostatic shift in heart rate (an increase of more than 20 beats per minute). A portion of the sample (*n* = 35) had bone mineral density results available, with 31.4% of the sample presenting with z scores equal to or less than − 1 for the lumbar spine and 34.3% for hip z-scores.

**Table 2:** Laboratory findings for male youth

	Measure of Central Tendency^a^, Sample size	Range	% Out of Range (reference range)
Metabolic panel			
Sodium (mmol/L)	141 (IQR = 3), 59	135–146	1.7% (135–145)
Potassium (mmol/L)	4.3 (SD = 0.42), 59	3.4–5.2	5.1% (3.5–5.0)
Chloride (mmol/L)	102 (IQR = 3), 50	95–111	4.0% (95–107)
Magnesium (mmol/L)	0.86 (SD = 0.08), 55	0.65–1.05	9.1% (0.74–0.99)
Phosphate (mmol/L)	1.37 (SD = 0.26), 56	0.22–1.95	23.2% (0.87–1.52)
Transaminases			
Aspartate aminotransferase (AST; U/L)	31 (IQR = 14), 44	15–112	25.0% (10–40)
Alanine aminotransferase (ALT; U/L)	27.5 (IQR = 23.75), 46	0–241	32.6% (10–45)
Cholesterol Panel			
Cholesterol (mmol/L)	3.65 (SD = 0.89), 24	1.3–5.2	16.7% (2.6–5.2)
Triglycerides (mmol/L)	0.70 (IQR = 0.72), 23	0.24–2.12	26.1% (0.4–1.5)
Hormones			
Testosterone (nmol/L)	6.1 (IQR = 14)^b^, 30	0.4–30.1^b^	13.3% (< 0.6)

^a^Mean (Standard Deviation) is reported for variables that were normally distributed. Median (Interquartile Range) is reported for variables with a non-normal distribution

^b^3 males had undetectable testosterone levels. The values presented are for those for whom hormone levels could be recorded by the laboratory (*n* = 27); however, the column for the percentage of participants for whom the values were out of range included those who had undetectable testosterone levels

**Table 3:** Medical characteristics of male youth

	Measure of Central Tendency^a^, Sample size	Range	% Out of Range (n; Reference Range)
Heart Rate			
Beats per minute (bpm, supine)	61.6 (SD = 18.5), 68	28 – 108	26.5% (18; < 50 bpm)
Orthostatic Shift (standing - lying)	16.5 (IQR = 15), 60	-6 – 87	33.3% (20; > 20 bpm)
Blood Pressure			
Systolic blood pressure (supine)	105.4 (SD = 12.8), 68	79 – 140	8.8% (6; < 90 mmHg)
Orthostatic shift in systolic pressure^b^	-1.54 (SD = 7.5), 63	-18 – 17	0% (0; > 20 mmHg drop)
Diastolic blood pressure (supine)	60.43 (SD = 8.6), 68	43 – 82	2.9% (2; < 45 mmHg)
Orthostatic shift in diastolic pressure^b^	-4.89 (SD = 7.4), 63	-24 – 11	22.2% (14; > 10 mmHg drop)
Bone Mineral Density (BMD)			*Reference Range:* *BMD z-score ≤ -1*
Spine z-score	-0.45 (SD = 1.0), 35	-2.4 – 1.9	31.4% (*n* = 11)
Hip z-score	-0.6 (SD = 1.2), 35	-3.0 – 2.1	34.3% (*n* = 12)
Total body z-score	-0.29 (SD = 1.2), 35	-3.2 – 2.1	28.6% (*n* = 10)

^a^Mean (Standard Deviation) is reported for variables that were normally distributed. Median (Interquartile Range) is reported for variables with a non-normal distribution

^b^Values for orthostatic shift in blood pressure are presented as lying-standing to examine postural drop. Reference range for heart rate and orthostatic shifts in pulse and blood pressure are based on admission criteria recommended by the Society for Adolescent Medicine [35]

### Sex differences: Youth treated at BC Children’s Hospital

Data from a subset of males who entered treatment after January 2010 were compared with data from biological females who were admitted to the inpatient or outpatient treatment streams at BC Children’s Hospital during the study period (2010–2015). A total of 41 males and 251 females were included in the dataset. In the female comparison group, 58 were treated in an outpatient setting (23.1% of female sample), and 193 were treated in the inpatient program (76.9% of female sample). In the male sample, 48.8% (*n* = 20) were initially treated in an outpatient setting, 12.2% (*n* = 5) in day treatment, and 39.0% (*n* = 16) in the inpatient program.

Due to unequal sample sizes and a violation of normality and/or homogeneity of variance assumptions, non-parametric tests were employed to assess sex differences in age at admission, duration of eating disorder symptoms, and age of onset. Male youth were significantly younger at admission than females (Mann-Whitney *U* = 3989.0, *z* = − 2.31, *p* = .021, *r* = − 0.14). The median age of males at admission was 14.9 years (IQR = 3.7, range = 9–17), and that of females was 15.5 years (IQR = 2.3, range = 9–18). No significant difference between males and females emerged for the duration of eating disorder symptoms at the time of admission (Mann-Whitney *U* = 4704.0, *z* = − 0.24, *p* = .81, *r* = − 0.01). Males reported a median duration of eating disorder symptoms of 11.0 months (IQR = 8.5, range = 2–96 months; *n* = 40) prior to admission, while females reported a median duration of 10.0 months (IQR = 12.0, range = 0–84 months; *n* = 241). The age of onset of eating disorder symptoms was calculated by subtracting the age at assessment by the duration of eating disorder symptoms. Data for the age of onset variable were normally distributed; however, there was a violation of homogeneity of variance. Given the large discrepancy in sample size between the two groups, non-parametric tests were performed, which demonstrated a significant sex difference, Mann-Whitney *U* = 2927.5, *z* = − 1.98, *p* = .047, *r* = − 0.13. Males had a younger age of onset (*M* = 13.1 years, *SD* = 2.7; n = 40) than did females (*M* = 14.2 years, *SD* = 1.6; *n* = 183). Females were more likely than males to come from a Caucasian ethnic background, *χ*2 (1, *N* = 243) = 11.36, *p* ≤ .001, odds ratio = 3.6 (CI = 1.7, 8.0). See Table 4 for details of sex differences.

**Table 4:** Sex differences in male and female youth who were treated at BC Children’s Hospital between 2010 and 2015

	Males, Sample Size	Females, Sample Size
Demographics	*n* = 30	*n* = 213
Ethnicity: Caucasian*	12 (40.0%)	151 (70.9%)
Ethnic Minority Group (includes Asian, East Indian, Aboriginal, mixed)*	18 (60.0%)	62 (29.1%)
Eating Disorder Diagnosis	*n* = 41	*n* = 251
Anorexia Nervosa or Bulimia Nervosa*	23 (56.1%)	208 (82.9%)
Atypical Anorexia Nervosa or Bulimia Nervosa*	10 (24.4%)	32 (12.7%)
Other Eating Disorder Diagnosis*	8 (19.5%)	11 (4.4%)
Eating Disorder Symptoms (Lifetime History)		
Binge-eating	13 (31.7%), 41	55 (22.4%), 245
Vomiting	9 (22.0%), 41	64 (26.2%), 244
Laxative Use	2 (4.9%), 41	25 (10.3%), 243
Excessive Exercise	28 (68.3%), 41	177 (72.8%), 243
Medical Characteristics		
Bradycardia (heart rate < 50 bpm)	9 (22.0%), 41	44 (23.9%), 184
Orthostatic shift in heart rate (> 20 bpm)	12 (32.4%), 37	61 (35.3%), 173
Bone Mineral Density – Spine z-score (≤ −1)	5 (22.7%), 22	75 (37.1%), 202

Note: This table does not capture all variables used for sex comparisons. Additional comparisons of variables not included in this table (i.e., treatment settings, age at admission, age at eating disorder onset, duration of eating disorder symptoms, and weight) are detailed in the results section

*denotes significant sex difference, *p* < .05

No significant differences in lifetime history of binge-eating (*p* = .20), vomiting (*p* = .56), laxative use (*p* = .27), or excessive exercise emerged (*p* = .55). Examination of sex differences in diagnostic presentation were performed, with youth classified into one of 3 groups: (1) diagnosis of AN or BN; (2) atypical AN or BN (including EDNOS with restrictive or binge-eating/purging symptoms); and (3) other eating disorder diagnoses (e.g., unspecified eating disorder, purging disorder, ARFID). Significant sex differences in diagnostic presentation emerged, *χ*2 (2, *N* = 292) = 18.91, *p* ≤ .001. Z-tests were used to compare column proportions, with a Bonferroni correction applied. Females were more likely than males to be diagnosed with AN or BN, whereas males were more likely to be diagnosed with an atypical eating disorder, or another eating disorder diagnosis. See Table 4 for details.

A subset of medical parameters was selected for assessment of sex differences, including heart rate and bone mineral density (see Table 4). The medical stability of males and females was similar, with no significant differences between sexes on the proportion of youth who met criteria for bradycardia (less than 50 beats per minute; *χ*2 (1, *N* = 225) = 0.07, *p* = .79), large orthostatic shift (greater than 20 beats per minute; *χ*2 (1, *N* = 210) = 0.11, *p* = .74), nor were there differences in the proportion of youth who had a bone mineral density z-score for the lumbar spine that was equal to or less than − 1, *χ*2 (1, *N* = 224) = 1.79, *p* = .18.

Females were more likely than were males to be underweight [less than 95% of mBMI; *χ*2 (1, *N* = 286) = 7.63, *p* = .006, odds ratio = 2.8 (CI = 1.3, 5.9)], with 85.7% of females (*n* = 210) and 68.3% of males (*n* = 28) classified as underweight at admission. Given a non-normal distribution of data for this variable, non-parametric tests were performed. Females who were underweight had a lower %mBMI at admission (Median = 78.0, *IQR = 12.1*) than did males (Median = 83.9, *IQR* = 11.0), Mann-Whitney *U* = 2020.0, *z* = − 2.69, *p* = .007, *r* = − 0.17). Females also had lost a larger percentage of weight [(maximum weight-admission weight)/maximum weight *100] than males, *F*(1,252) = 4.67, *p* = .032, partial η^2^ = .018. Females lost an average of 20.3% of their maximum body weight (SD = 10.1, CI = 18.9, 21.7; *n* = 217), whereas males lost an average of 16.3% (SD = 11.9, CI = 12.9, 19.7; *n* = 37).

## Discussion

The males who presented to our service demonstrated significant medical compromise, despite being only moderately underweight (with an average of 87.5% mBMI). Vo and colleagues [14] reported a similar clinical presentation of males, with a mean of 88% mBMI in their sample. As with Vo and colleagues, a portion of the sample presented with indices of medical instability, including very low heart rate (26.5%) and a large orthostatic shift in heart rate (33.3%). Bone mineral density results were available for a portion of the males included in the chart review. Approximately one-third of males presented with bone mineral density z-scores equal to or less than − 1 (with 31.4% presenting with low z-scores for the lumbar spine and 34.3% with low z-scores for the hip), which is similar to the report by Norris and colleagues [13] that 39% of males had osteopenia or osteoporosis based on z-scores for the lumbar spine. Similarly, previous reports indicated that 35% of male adolescents with AN presented with results consistent with a diagnosis of osteopenia [39]. Testosterone levels were low in 13% of the sample, including 3 males for whom testosterone was undetectable. Previous reports have highlighted diversity in endocrinopathies in a case series of adult males with AN [40]. Testosterone levels in males with AN have been demonstrated to rise with weight gain, though the rate of increase in testosterone levels varied across individuals [41]. Given that testosterone values were available for only 42% of the total sample (*n* = 30) in the current study, it was not possible to evaluate the relationship between testosterone levels and % mBMI. One of the factors driving the lack of availability of hormone levels (and other biochemistry investigations) for a portion of the sample was that a standardized process for biochemistry investigations was not in place in the Eating Disorders Program until 2011, and was also not yet in place at Woodstone (now known as Looking Glass Residence) at the time of assessment for the male residents.

The current results extended previous findings reported by Kinasz and colleagues [26] and Shu and colleagues [28], in demonstrating a higher prevalence of atypical and other eating disorder diagnoses in males than in females. Males in the current study were also less likely to be diagnosed with AN or BN. Kinasz and colleagues [26] have suggested that the diagnosis of an other specified eating disorder may represent a “catch all” category for males (p. 415). The higher prevalence of atypical eating disorder diagnoses may be accounted for in part by the lower percentage of weight loss in males observed in the current study. Males were also more likely to come from an ethnic minority group than were females, further substantiating the report by Kinasz and colleagues [26]. The diversity in ethnic background in our sample of males is reflective of the population in the area served by the hospital, with 51.8% of the population in Vancouver belonging to a visible minority group (and 27.3% of individuals in the province of British Columbia belonging to a visible minority) [42]. Also supporting the hypotheses was the younger age of onset in the male sample; however, no significant differences emerged in the duration of eating disorder symptoms.

The medical stability of male and female youth was similar, which mirrors previous findings [28]. There were no sex differences in the proportion of males and females who had an abnormal z-score for the lumbar spine from the bone mineral density assessment. Past research has suggested that males with AN have more pronounced bone loss than do females [22]; however, this research included adult males in the sample. We also examined sex differences in body weight at presentation. Given the higher proportion of females who were underweight at admission, we limited this analysis to those who were underweight. As in previous research (e.g., [15]), males presented at a higher % mBMI than did females.

The diagnostic distribution of males included a portion of individuals who did not meet full criteria for an eating disorder, as well as a significant minority of the sample who met criteria for EDNOS/OSFED. Males were less likely than were females to be diagnosed with AN or BN. Previous studies with male adolescents have reported mixed results. Some studies report that atypical diagnoses are the most common diagnoses of males presenting for eating disorder treatment (e.g., [6, 13]), while others report that anorexia nervosa is the most common diagnosis (e.g., [4]). A previous report on a subsample of males treated in our inpatient eating disorders program also found AN to be the most common diagnosis [32]. We hypothesized that the presentation of males with an eating disorder diagnosis other than AN or BN may be more common in outpatient programs, and the results of this study partially support this prediction. Males with a diagnosis of AN or BN were more likely to be treated in intensive (inpatient/day treatment) services, while those with an other eating disorder diagnosis were more likely to receive treatment in outpatient services. There were no differences in the proportion of males with a diagnosis of atypical AN or BN diagnosis across the intensive and outpatient services. Given the differences in diagnostic presentation across services, it is important to consider the intensity of treatment when comparing the clinical characteristics across samples of males with eating disorders. This also suggests that the results regarding the more common prevalence of atypical and other eating disorder diagnoses in males need to be interpreted with caution, given that the majority (76.9%) of the female sample was from an inpatient treatment setting, whereas only 39% of the 41 males who were included in the analyses on sex differences were initially treated in the inpatient setting.

One of the limitations of this study is that the dataset of females does not represent all the individuals who were treated in the program over the time period, but rather a convenience sample of a portion of females (treated in outpatient family-based therapy or inpatient) for whom data were available. Further prospective research investigating sex and gender differences across treatment intensity is warranted to clarify whether the increased prevalence of atypical eating disorder diagnoses in males, as reported in Shu et al. [28], are found across the continuum of care.

There were several variables for which there were non-normal distributions of the data, both in clinical presentation (e.g., duration of eating disorder symptoms) as well as medical characteristics (e.g., testosterone levels, and measures within the metabolic panel). Therefore, there appears to be a heterogeneity in males who present to a tertiary pediatric eating disorder service. The non-normal distributions led to use of non-parametric tests to detect sex differences, which have a lower relative power than would a parametric test (assuming the underlying assumptions of the test can be met) [43]. Furthermore, due to relatively small sample sizes across the diagnoses, we were not able to examine differences across each of the eating disorder subtypes. Furthermore, given the long study period, there were changes in eating disorder diagnostic criteria from DSM-IV-TR [36] to DSM-5 [29]. We grouped together diagnostic presentations (e.g., considering whether those with an EDNOS diagnosis presented with symptoms of restriction or binge-eating/purging), to facilitate comparison of diagnoses across the study period. However, due to a lack of detail in the frequency and duration of symptoms noted in the older assessment notes that were examined as part of this retrospective chart review, it was not possible to reclassify DSM-IV-TR diagnoses into DSM-5 diagnoses. It is therefore not possible to confirm whether some of the other diagnoses reported in the sample (e.g., food avoidance emotional disorder) would now meet criteria for ARFID. Given the report by Vo and colleagues [14] that there was a decrease in the prevalence of EDNOS/OSFED diagnosis when applying DSM-5 criteria, it will be important to continue investigating the pattern of diagnostic presentation in adolescent males.

Strengths of the current study include the large sample size of 71 male children and youth for the descriptive analyses, the wide range of physical assessments, and the detailed psychiatric assessments that were obtained as part of clinical practice. Self-report tools to assess eating disorder and related symptoms were not included in the current study. Males have reported less severe eating pathology than do females (e.g., [26, 28]), as measured by the Eating Disorder Examination [44]. However, the most common measures in the field of eating disorders have been developed to assess female concerns, and recommendations have been put forth by Darcy and Lin [45] regarding tools that include male-specific concerns, including muscularity. A recent review by Murray and colleagues [7] highlighted the lack of focus on muscularity concerns in current diagnostic criteria for eating disorders, and suggests that changes may be necessary to the conceptualization of eating disorder pathology to be more inclusive across the gender spectrum. Our group has undertaken a prospective study of all biological males who are admitted to our service (along with a group of matched females), to follow-up on some of the observed sex differences in pediatric eating disorder presentation. We have included validated measures designed to assess concerns that may be more relevant for males, such as muscularity, in accordance with the recommendations of Darcy and Lin [45].

## Conclusion

Some consistent sex differences in pediatric eating disorders appear to be emerging, including males having a younger age of presentation, younger age of onset, a higher body weight at admission (as measured by %mBMI), and a lower prevalence of AN or BN eating disorder diagnoses. There appear to be significant medical sequelae in male children and youth with eating disorders, despite the smaller percentage of weight loss relative to females for those who were underweight at admission.

## Abbreviations

% mBMI: Percentage median body mass index
AN: Anorexia Nervosa
ARFID: Avoidant/Restrictive Food Intake Disorder
BC: British Columbia
BMI: Body Mass Index
BN: Bulimia Nervosa
CI: Confidence Interval
DSM: Diagnostic and Statistical Manual of Mental Disorders
EDNOS: Eating Disorder Not Otherwise Specified
IQR: Interquartile Range
OSFED: Other Specified Feeding or Eating Disorder
REDCap: Research Electronic Data Capture
SD: Standard Deviation

## Footnotes

1 This group of participants represents an expansion of a previous sample of male youth (*n* = 23, who were admitted to inpatient treatment) reported in Coelho et al., 2015 [32]

2 A portion of this sample (females with AN who were treated between 2010 and 2014) were included in a report by Janzen and colleagues (Janzen M, Cheung C, Steinberg C, Lam PY, Krahn A: Changes on the electrocardiogram in anorexia nervosa: a case control study, submitted). The current sample was expanded to include females who were admitted to the inpatient unit (March 2010 – June 2015) across all eating disorder diagnoses

3 A portion of this sample was included in a report by Coelho and colleagues (Coelho JS, Beach B, O’Brien K, Marshall S, Lam PY: Effectiveness of family-based treatment for pediatric eating disorders in a tertiary care setting, submitted). Only biological females who were admitted during the study period (2010–2015) were included in the analyses for the current study
